# Tuneable strong optical absorption in a graphene-insulator-metal hybrid plasmonic device

**DOI:** 10.1038/s41598-017-07254-0

**Published:** 2017-08-04

**Authors:** N. Matthaiakakis, Xingzhao Yan, H. Mizuta, M. D. B. Charlton

**Affiliations:** 1 Department of Electronics and Computer Science, University of Southampton, Southampton, SO17 1BJ United Kingdom; 2 School of Materials Science, Japan Advanced Institute of Science and Technology, Ishikawa, 923-1292 Japan

## Abstract

An optical device configuration allowing efficient electrical tuning of near total optical absorption in monolayer graphene is reported. This is achieved by combining a two-dimensional gold coated diffraction grating with a transparent spacer and a suspended graphene layer to form a doubly resonant plasmonic structure. Electrical tuneability is achieved with the inclusion of an ionic gel layer which plays the role of the gate dielectric. The underlying grating comprises a 2-dimensional array of inverted pyramids with a triple layer coating consisting of a reflective gold layer and two transparent dielectric spacers, also forming a vertical micro-cavity known as a Salisbury screen. Resonant coupling of plasmons between the gold grating and graphene result in strong enhancement of plasmon excitations in the atomic monolayer. Plasmon excitations can be dynamically switched off by lowering the chemical potential of graphene. Very high absorption values for an atomic monolayer and large tuning range, extremely large electrostatically induced changes in absorption over very small shifts in chemical potential are possible thus allowing for very sharp transitions in the optical behavior of the device. Overall this leads to the possibility of making electrically tunable plasmonic switches and optical memory elements by exploiting slow modes.

## Introduction

Graphene is an atomic monolayer consisting of carbon atoms tightly packed in a two-dimensional honeycomb lattice. As a promising newly discovered material combining remarkable electronic, photonic and mechanical properties graphene has attracted a lot of attention in the scientific community^1–6^. The demonstrated high quantum efficiency for light matter interactions as well as its strong optical nonlinearity, high optical damage threshold, and most importantly the ability to easily tune its optical properties, promotes graphene as a highly promising candidate for photonic devices^5^. Nevertheless, even though 2.3% of optical absorption is impressive for an atomic monolayer, it is not enough for real world applications which require much higher absorption values for efficient operation. Enhancing light absorption in graphene while maintaining the ability to have strong and dynamic switching or efficient frequency modulation of this absorption is crucial for the commercialization of graphene based active photonic devices.

Excellent progress has recently been made to achieve enhanced light absorption in graphene. Perfect graphene absorbers have been proposed based on graphene disks^7^, and ribbons^8^, positioned a small distance from a metallic ground plate. These take advantage of interference effects provided by the spacer geometry helping to dissipate incoming radiation. Additionally, patterned graphene has been used as a light trapping component that can enhance optical absorption in surrounding absorptive mediums^9^. Attenuation total reflection multilayer structure configurations (prism/graphene/Quartz) have also been used to significantly enhance light absorption in graphene at optical frequencies^10^. In such devices coherent absorption is achieved by utilizing interference effects in a multilayer structure giving rise to a large electric field intensity in the vicinity of the graphene layer. Hetero-structures devices have also been theoretically suggested to achieve perfect absorption in the THz region by taking advantage of the strong photon localization in an introduced graphene defect layer^11^. Furthermore, graphene/MgF_2_ multilayer stack unit cell arrays on Au film plane have been theoretically predicted to achieve a dual-band strong absorption effect^12^.

Devices implementing a Salisbury screen^13^ have also been proposed to enhance optical absorption in graphene since they can provide strong field enhancement in the vicinity of the monolayer when carefully optimized. Such devices have effectively been used to enhance interband absorptions in graphene by a factor of 5.5 in the mid-infrared spectral region, also providing a very strong modulation efficiency of 3.3% of absorption change per Volt but provide limited amount of absorption enhancement and wavelength modulation^14^. It has been theoretically predicted that under grazing angle for s polarized incident light such Salisbury screen/graphene setups can provide nearly total optical absorption, also achievable when using multiple stacked graphene films, but wavelength tuning remains weak^15^. Absorption due to plasmon excitations in graphene nano-resonators have also been suggested to be enhanced when utilizing Salisbury screen setups, also allowing for absorption tuning by electrostatic gating, but the absorption efficiency remains low (24.5%)^16^.

Hybrid metal-graphene structures have been studied by several independent groups demonstrating strong optical absorption enhancement in graphene. Theoretical studies have shown that ribbon^17^ or cross-shaped^18, 19^ metallic resonators positioned above a graphene layer can, at certain resonant conditions, be used as light trapping components for increasing the interaction of light with graphene, thus achieving strong optical absorption. Similarly cross and ribbon shaped metallic resonators combined with double layer graphene wires have been theoretically studied achieving similar results^20^. Furthermore, metamaterial resonators like split rings^21^ and interdigitated structures^22^ have been combined with single or multilayer graphene stacks providing high speed modulation capabilities as well as strong absorption modulation. Theoretical and experimental studies where graphene is used to fill several periodic metallic apertures of subwavelength sizes have also demonstrated high optical absorption^23, 24^. Finally critical coupling with photonic crystal slab guide mode resonances has been predicted to achieve strong absorption^25^.

Nevertheless, achieving strong optical absorption in monolayer graphene in combination with the ability to dynamically tune the wavelength of absorption over a large spectral range and especially away from mid infrared and THz frequencies remains a difficult but highly anticipated task. In this work, a device capable of overcoming these limitations by providing efficient electrical modulation of nearly total optical absorption over a large range of wavelengths while allowing operation even at Mid-infrared and almost visible light frequencies is presented.

**Figure 1:**
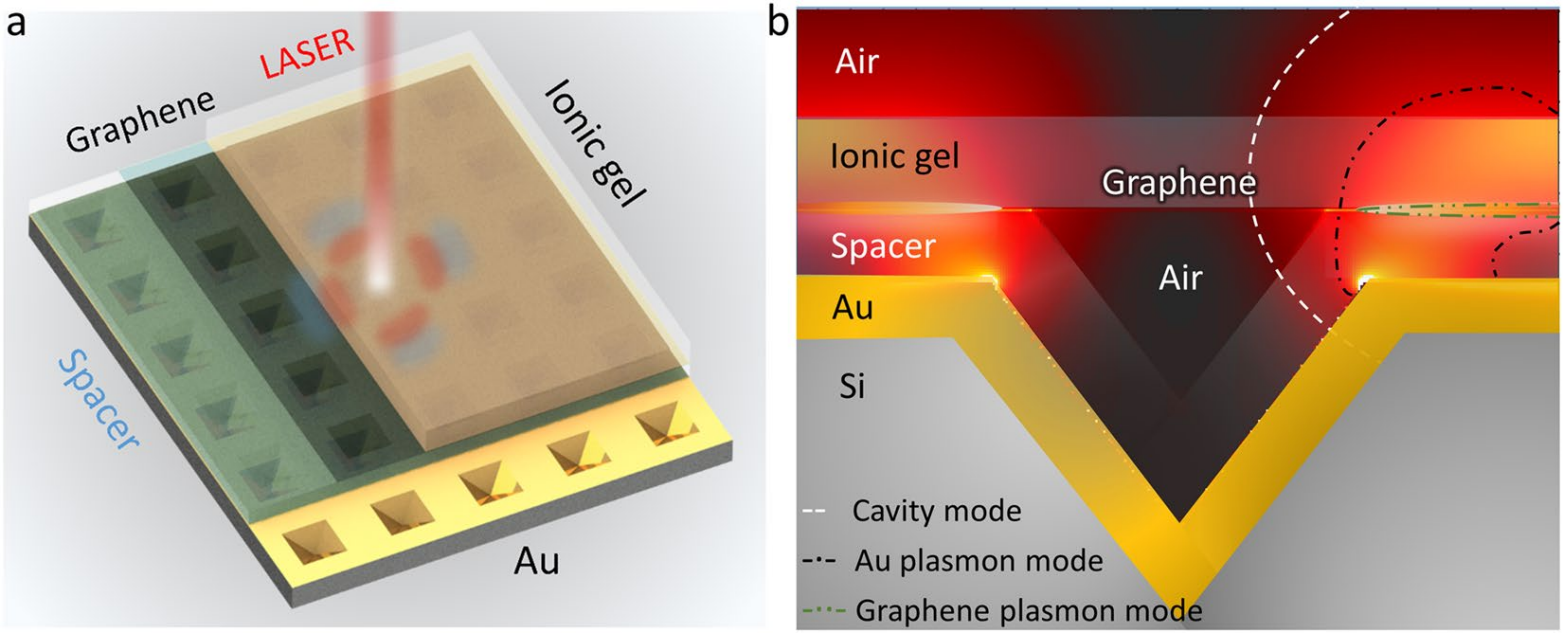
(**a**) Schematic of the device setup. (**b**) Artistic representation of the device operation showing the different electromagnetic field components in the device.

Figure 1a shows a schematic of the device. A two-dimensional array of inverted pyramid pits forms a crossed diffraction grating which functions as a phase-matching component that couples incident photons to plasmons in a continuous graphene layer^26^. In contrast to previous works, a continuous graphene layer, instead of micro/nano patterned graphene, allows excitation of propagating modes instead of localized modes. Such propagating modes typically have higher field confinement in the surface normal^27^.

Optical absorption by the monolayer is further enhanced by positioning Graphene in close vicinity to a gold layer that also allows propagation of plasmons. The structure consists of a conformal two-layer metal-dielectric coating deposited over the underlying diffraction grating. The role of the dielectric spacer is to separate the graphene layer from the gold back reflector but can also be used as a back-gate in order to modulate the chemical potential of graphene. An ionic gel layer is further placed on top of graphene in order to provide a highly efficient gating method for achieving strong electrostatic doping of the atomic monolayer (there is a large variety of ionic gel types that can be used^28^). Using an ionic gel as the top dielectric overcoat material in a gate setup^28, 29^ enables strong modulation of the chemical potential at low voltage operation (from 0 eV up to 0.8 eV within 3 V of applied voltage)^30^ thus providing a wide wavelength tuning range for plasmon excitations in the graphene layer. The inclusion of the spacer and ion gel layers also results in the formation of a weak 1-dimensional micro-cavity transverse to the grating surface that is also known as a Salisbury screen^13^.

When phase matching conditions are met, incident light couples by diffraction to slowly propagating surface plasmon modes (traveling across the micro-structured surface), associated with the periodic gold coated pyramid structure. Similarly, surface plasmon modes are excited on the suspended graphene layer. Due to the geometry of the structure and the excitation of the gold plasmon mode, at the edges of the pyramid groove a strong near field is produced that is extended upwards and strongly interacts with the plasmon mode in graphene (Fig. 1b). This results in a doubly resonant mode and in the transfer of energy from the gold plasmon mode to the free carriers in graphene thus indirectly increasing coupling of light to the atomic monolayer. Stationary modes of the vertical Salisbury screen cavity do not directly transfer energy to the plasmon modes but independently increase the total amount of absorption in the device. This two-step energy transfer process is explained and validated in full detail later. Overall this arrangement results in strong enhancement of light absorption in the monolayer, greatly increasing coupling efficiency between incident light and graphene.

Most importantly the resonance frequency can be adjusted by changing the chemical potential of the graphene layer. Using realistic chemical potentials for the graphene layer it is demonstrated that optical absorption can be tuned from mid infrared frequencies to near-infrared wavelengths (up to 1.4 μm for a chemical potential of 0.65 eV). The rate of wavelength tuning for plasmon absorption was found to be 100 nm/0.05 eV of change in graphene chemical potential (estimated 1 μm/0.5 V at the near infrared region).

Rigorous coupled-wave-analysis (RCWA) simulations allowed step by step analysis of the multi-stage plasmon coupling mechanisms, confirming strong enhancement of graphene plasmon modes over a spectral range of a few hundred nm.

The wavelength of the gold plasmon peak as well as of the diffraction and Salisbury screen features can be determined by geometric parameters of the pyramid structures and of the vertical micro-cavity such as: spacer and ionic gel layer thickness and index of refraction. Optimization of these parameters yielded 1650% increase in absorption of incident light by the graphene mono-layer, compared to a non-resonant device (not implementing the proposed graphene-gold hybrid structure), while maintaining a large tuning range (defined by the geometrical and structural parameters of the device) and excellent electrical modulation efficiency.

Since the graphene absorption peak is spectrally very narrow and electrical gate control voltage low, our configuration potentially allows rapid dynamic switching between high and low absorption states by electrical modulation, providing a new device for fast plasmon switching in planar light circuits (PLC). Plasmon frequency can be also adjusted electrically by the gate arrangement, making it suitable for tunable chemical sensors and photonic circuits.

### Exciting and tuning plasmons in graphene

Graphene is an atomic monolayer consisting of carbon atoms arranged in a honeycomb lattice formed by sp^2^ bonds^5^. The band structure of graphene is formed by overlapping p_z_ orbitals of neighboring carbon atoms. Bonding π states form the valence band, and the antibonding π* states form the conduction band giving a linear dispertion^5, 6^ for energies bellow ~1.5 eV. Conduction and valance bands touch at six points known as the Dirac points and can be very accurately approximated as two symmetric cones for low energies. For undoped graphene the Fermi level lies exactly on the Dirac point (effectively at the intersection between the valence and conduction bands) and so there is a very small number of free electrons. This in combination with the lack of a band gap allows for direct dynamic tuning of the carrier concentration and position of the chemical potential through electrostatic gating^31^. Electrostatically tuning the carrier concentration in graphene allows for direct control of complex permittivity of the monolayer. Since plasmon dispersion is directly related to the relative permittivity of the conductor in comparison to that of the surrounding dielectric material, direct electrostatic control of Plasmons is possible.

**Figure 2:**
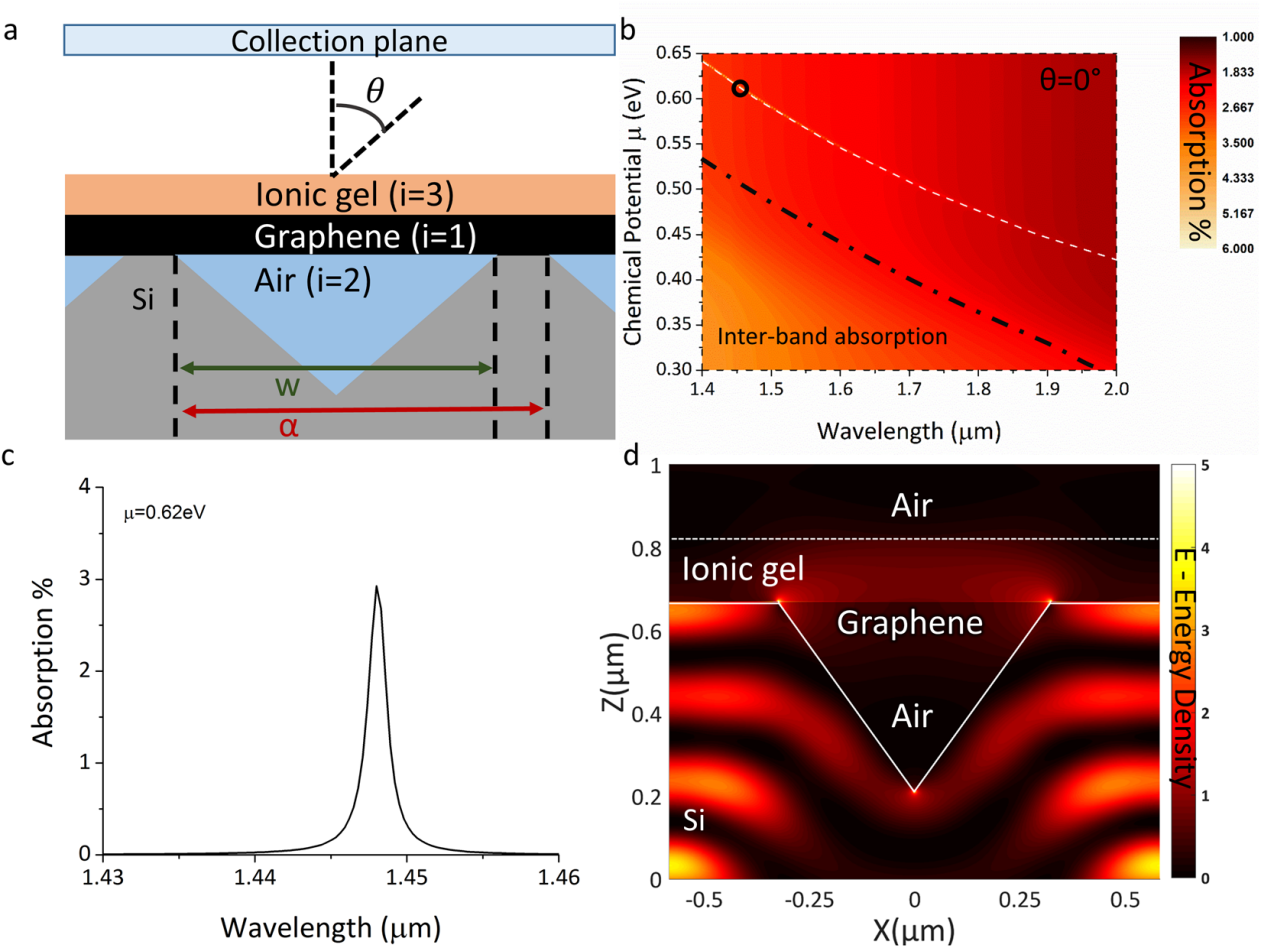
(**a**) Slice schematic of the 2-D inverted pyramid diffraction grating (**b**) RCWA simulation results of the same device demonstrating excitation and electrostatic control over plasmons in graphene. (**c**) Plasmon absorption for a chemical potential of 0.62 eV. (**d**) FDTD simulation at a wavelength of 1.448 μm and P polarization demonstrating the E field density resulting from the interaction of incident radiation with the diffraction grating, chemical potential/Wavelength combination corresponds to the black circle of b.

Under normal conditions incident photons cannot couple to and excite plasmons due to the wave vector of plasmons being higher than that of photons for any given frequency. Mode-matching techniques can be used to provide phase matching between incident photons and plasmons. In this case a complex underlying 2D inverted pyramid diffraction grating is used for coupling plasmons on the graphene layer (Fig. 2a shows a slice schematic of the structure)^26^. Phase matching conditions (and thus the excitation of plasmons) can be estimated by assuming a 1D grating structure.

1 $${k}_{sp}={k}_{x}\pm {v}_{o}2\pi /\alpha $$

where *k*_*sp*_ the wave vector of plasmons in Graphene, grating order *v*_*o*_ is an integer (1, 2, 3,…), *α* the pitch of the grating structure, and *k*_*x*_ = *ksinθ* the in plane wave vector of impinging photons.

Figure 2b shows spectra generated by RCWA analysis for a single graphene layer positioned above a silicon 2-D inverted pyramid diffraction grating with 1.165 μm pitch, pyramid base width of 0.65 μm, ionic gel refractive index of 1.42 (as reported in refs 32 and 33) and thickness 150 nm, as a function of chemical potential for an angle of incidence of θ = 0°. It can be seen that plasmon absorption can be tuned over a large wavelength range by adjusting chemical potential. Solutions to equation 1 are superimposed as a white dashed line overlaying perfectly with the RCWA data. Inter-band absorption can be observed as a broad absorption region when moving to higher frequencies and lower chemical potentials since the Pauli blocking effect is no longer evident (region below the black dash-dot line). Nevertheless, from Fig. 2c it is clear that the absorption efficiency for this structure is very poor with only about 3% of incident light absorbed by the graphene layer.

Figure 2d shows an example FDTD simulation plotting E-field component perpendicular to the diffraction structures for a wavelength/Chemical potential combination corresponding to the black circle marker of Fig. 2b. It can be seen that a large part of the incident field is lost in the bulk silicon material due to its transparency in the infrared, and so does not assist the generation of graphene plasmons.

### Strong enhancement of optical absorption in graphene with the introduction of hybrid gold-insulator-graphene multilayer structure

In our device, optical absorption of the graphene layer can be enhanced by coating the grating with a conformal reflective gold layer and a transparent dielectric (SiO_2_, or ITO) spacer layer, with the absorbing graphene mono-layer placed on top. This setup allows for transfer energy from the excited gold plasmon mode to the graphene plasmon mode, thus increasing the efficiency for coupling light in the atomic monolayer. Once again an ionic gel layer serves the role of the transparent gate dielectric for controlling the chemical potential of graphene. The inclusion of the spacer and ITO layers also forms a flexible vertical Salisbury screen micro-cavity (more information in the supplementary document).

### Simulation Results

**Figure 3:**
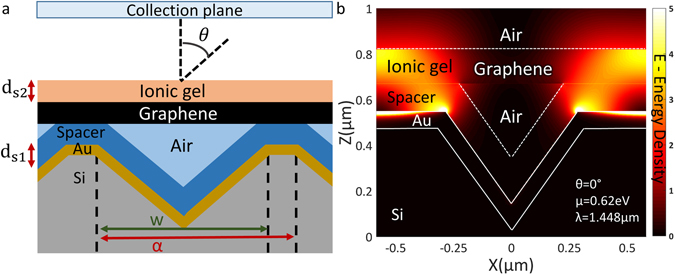
(**a**) Schematic setup and (**b**) E field density plot for the hybrid gold-insulator-graphene plasmonic device.

Identical parameters for the diffraction grating and ionic gel as used for the non-hybrid structure in Fig. 2 are now used for FDTD and RCWA simulations of the multilayer structure (*stack layers: 75 nm Gold*, *120 nm SiO2*, *graphene*, *150 nm ion gel*) (Fig. 3a).

As seen in Fig. 3b the gold back reflector no longer allows the radiation to leak through the silicon substrate, significantly enhancing the diffraction efficiency of the pyramid structures. It is clear from Fig. 3b that the graphene layer can be moved across the vertical cavity by adjusting the spacer/ion gel thickness ratio. (*Detailed discussion of Fig.* 3b *is given later*).

**Figure 4:**
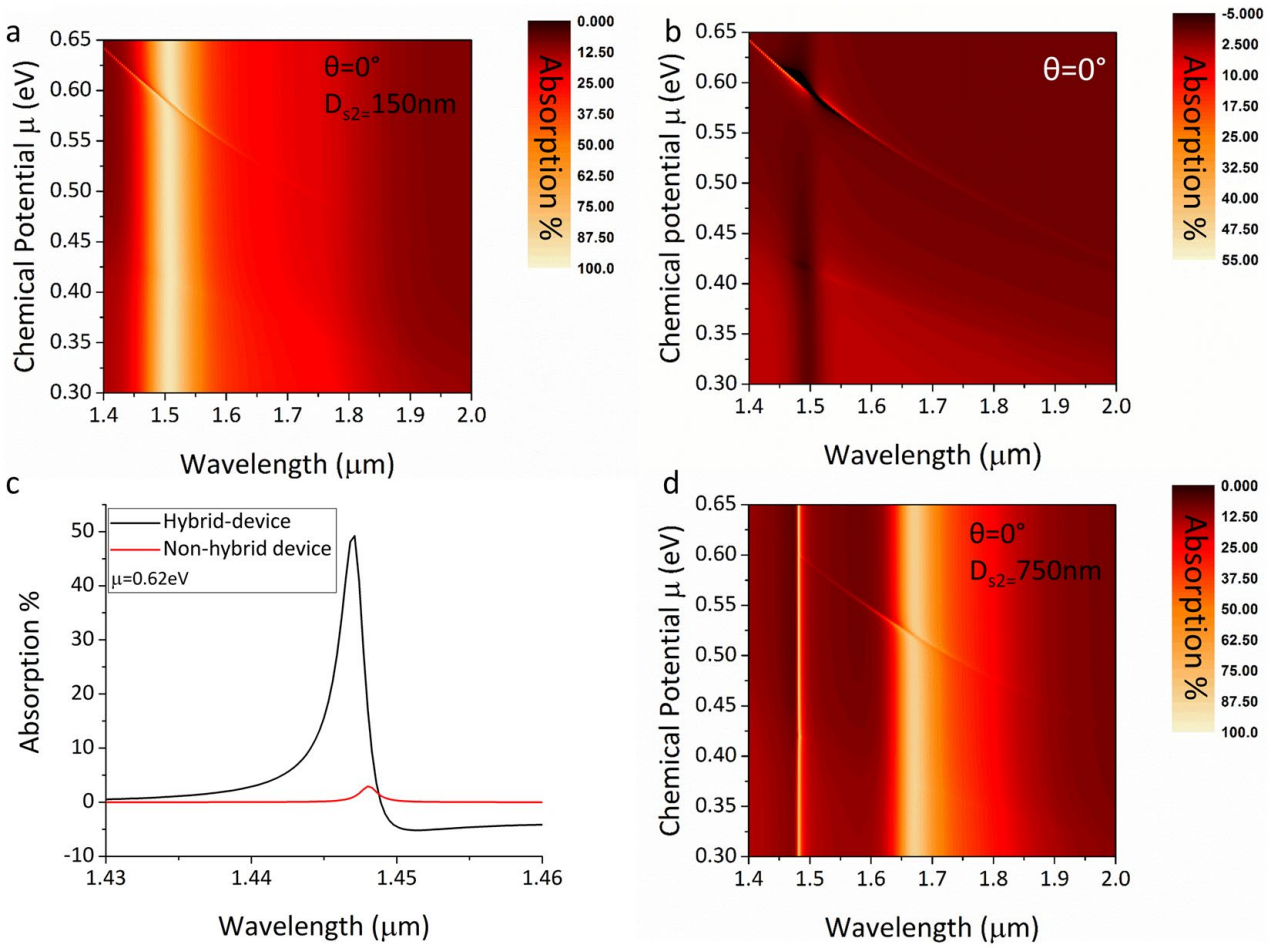
RCWA simulation spectra for the hybrid device setup where large tuneable optical absorption approaching 100% can be observed. (**a**) Resulting spectra for a device with 150 nm ionic gel thickness. (**b**) Absorption attributed only to the graphene layer for the same setup by subtracting absorption due to the grating structure and other layers. (**c**) Comparison of absorption between non-hybrid and hybrid device. (**d**) Resulting spectra for a device with an ionic gel thickness of 750 nm.

Figure 4a shows simulated spectra (plotted as a colour map) for a range of chemical potentials. The broad absorption peak centred at 1.5 μm is independent of chemical potential and is due to the excitation of a gold plasmon mode. The sharp diagonal curved line which has a strong dependence on the chemical potential of the graphene layer is due a plasmon mode excited in graphene.

From the contour plot in Fig. 4a, it is clear that when the graphene and gold plasmon modes overlap there is a transfer of energy to the graphene plasmon mode and the absorption in the atomic monolayer is significantly increased. This is verified by the FDTD simulation result in Fig. 3b where it can be observed that there is a strong near field originating from the gold plasmon mode that is projected upwards from the edges of the pyramid structure and strongly interacts with the graphene layer. Near 100% tuneable absorption occurs at wavelengths where the graphene plasmon peak is in close vicinity to the gold plasmon peak while strong absorption (60–100%) occurs over a bandwidth of a few hundred nm when the graphene plasmon peak is tuned further away. It is thus clear that this doubly resonant process provides strong dynamically tunable optical absorption.

It is important to mention that in this case the Salisbury screen cavity mode has been tuned to overlap with the gold plasmon mode. Due to the inclusion of the pyramid structures and the limited amount of flat area on the surface of the device the vertical Salisbury screen cavity only contributes to a few percent of the total optical absorption from the device and thus it is difficult to observe from this graph. The absorption of the Salisbury screen has been identified as an isolated absorption mechanism in the device and does not contribute in energy transfer towards the graphene layer. More information about the absorption due to the Salisbury screen cavity is provided later on.

Absorption attributed solely to the graphene layer can be de-convolved from the data by subtracting equivalent results for the multilayer structure without the graphene layer. This is plotted in Fig. 4b and reveals an impressive 50% transfer of power to a single graphene monolayer. For shorter wavelengths and lower chemical potentials, the inter-band absorption in graphene is still visible over a broad spectral range. By selecting the data for a specific chemical potential value from Fig. 4b and ccomparing the resulting spectra with those of the non-hybrid device, a boost in graphene layer absorption of 1650% can be observed (Fig. 4c).

The enhancement wavelength range can be tuned by changing the thickness or refractive index of either of the transparent layers (spacer/ionic gel). An example of this can be seen in Fig. 4d where increasing the thickness of the ion gel layer to 750 nm shifts the enhancement range by almost 0.2 μm.

It is important to mention that the modulation of plasmon frequency is very strong, demonstrating a wavelength shift of about 100 nm per 0.05 eV of chemical potential change in graphene (estimated 1 μm/0.5 V at the near infrared region - more details can be found in the supplementary information) allowing for extremely low voltage tuning of absorption over a broad spectral range. Combining strong absorption and effective dynamic tuning over a large spectral range is ideal for fabrication of highly efficient modulators. Additionally the plasmon excitation can be effectively shut down by lowering the chemical potential (voltage) in graphene thus providing capability for dynamic electro-optical switching.

### Deconvolution and explanation of plasmon coupling mechanisms

**Figure 5:**
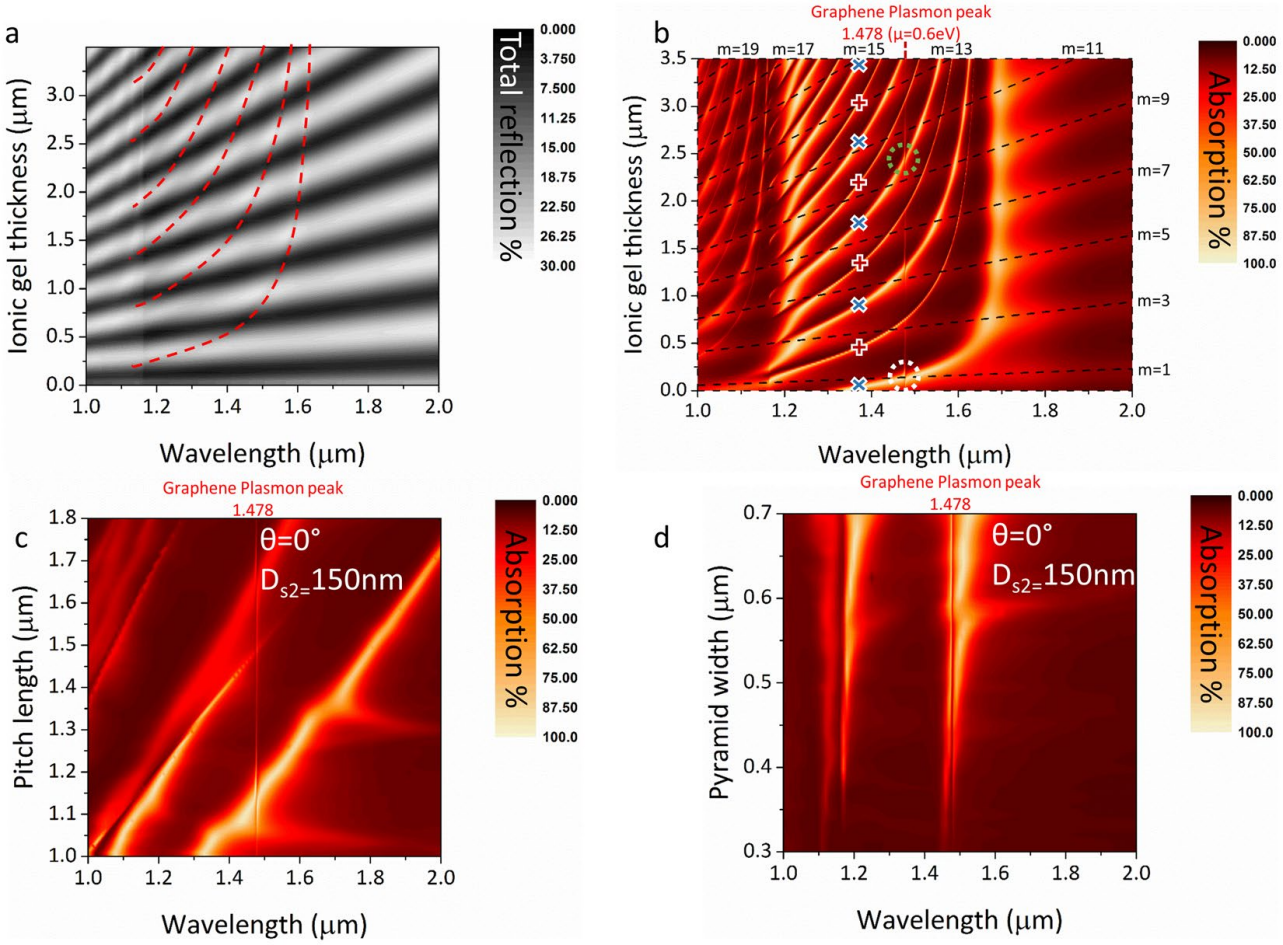
RCWA simulation of the device showing (**a**) Total reflection spectra for varying ionic gel thickness when not including the gold back reflector and graphene layer, red dashed lines correspond to dispersion modes of the pyramid grating structure (**b**) Absorption spectra now including the graphene and Au layers, vertical absorption line corresponds to plasmon excitations labelled at 1.478 μm, the black dashed lines correspond to destructive interference originating from the micro-cavity, curved lines marked with blue “x” markers to Au plasmon modes and curved lines marked with red “+” markers to modes of the pyramid grating structure. Green and white dashed circles are examples of doubly resonant modes. (**c**,**d**) correspond to RCWA simulation results for varying pitch lengths and pyramid structure size respectively.

We now de-convolve the physical processes underway on a step by step basis. Figure 5a plots total reflection of normally incident light as a function of ion gel thickness for a simplified structure consisting of the underlying grating coated with the 2-layer dielectric stack, but without the gold back reflector or graphene layer. This is equivalent to a fully dielectric structure supporting photonic crystal/diffraction modes associated with the lateral grating, (propagating across the surface of the device), as well as slow/static modes in the vertical direction associated with the cavity formed by the dielectric bi-layer coating. In contrast to Figs 2 and 3, total reflection is plotted instead of absorption in order to enhance visibility of key features which are much weaker due to the exclusion of the gold layer.

The set of sharp (but very feint) curved lines (labeled with red dashed lines) correspond to dispersion modes associated with the underlying dielectric pyramid grating structure. These relate to phase matching conditions coupling vertically incident light from free space to lateral propagating modes in the dielectric structure via a scattering/diffraction mechanism^34^.

The dark fringes result from destructive interference in the dielectric bi-layer stack, whereas the broad light fringes are associated with weakly resonant modes of the vertical micro-cavity resulting from constructive interference (see Salisbury screen in the supplementary information). The conditions for destructive interference in this vertical cavity are:

2 $$\lambda =4({n}_{s1}{d}_{s1}+{n}_{s2}{d}_{s2})/m$$

where d_s1_ the thickness of the spacer and n_s1_ the value of the spacer refractive index, d_s2_ the thickness of the ion gel and n_s2_ the refractive index of the ionic gel, and m is an integer cavity mode number.

Figure 5b plots data for the full structure now including the gold back reflector and graphene layers, for a fixed graphene chemical potential of 0.6 eV. Solutions of equation 2 are overlaid for a range of values of d_s2_ as black dashed lines revealing perfect agreement with the diagonal bright fringes seen in the RCWA simulation. Hence Fig. 5b reveals how variation in ionic gel layer thickness affects wavelength conditions for destructive interference in the vertical dielectric cavity and its interaction with diffraction conditions associated with the pyramid grating. As the vertical micro-cavity becomes wider (becomes larger) destructive interference conditions shifts to longer wavelengths. Eventually the cavity becomes multi-moded and destructive interference conditions become satisfied for more than one value of *m*, hence the fringes become periodic with cavity width.

With the inclusion of the gold back reflector the diffraction lines previously associated with the dielectric grating (marked with red crosses in Fig. 5b) become very sharp and visible. More significantly, an extra set of dispersion lines appears in-between them. These correspond to surface plasmon mode dispersion associated with the periodic gold coating (marked with blue × symbols)^35^.

Introduction of the graphene layer gives rise to a sharp vertical line at 1.478 μm corresponding to plasmon modes supported by the graphene monolayer. Points where the graphene resonance line crosses the gold plasmon mode dispersion features (curved lines marked with x) correspond to resonant coupling conditions (perfect phase matching) between plasmons supported by the underlying periodic gold pyramid structure, and surface plasmons supported by the graphene film (green dashed circle marker). Energy transfer to the graphene layer, and thus higher absorption in the monolayer, was found to occur only when the graphene plasmon peak overlaps or is in close vicinity to the gold plasmon peaks.

On the contrary, when the graphene peak overlaps with diffraction features (marked with red crosses), or features originating from the Salisbury screen vertical cavity (marked by black dashed lines), the amount of absorption attributed to the graphene monolayer is not increased. When features overlap with the Salisbury screen peak the overall absorption from the device increases (as marked by the white dashed circle marker). It is important to mention though that this increase is a simple additive effect and there is not transfer of energy between the individual effects and the vertical micro-cavity mode (see supplementary information and Figure s3 for more details). For transfer of energy to occur, not only the intensity but also the gradient of the field is important.

The wavelength of dispersion features associated with the underlying grating structure (curved lines labelled with + and × markers in Fig. 5b) depend on the pitch and size of the pyramid structures, providing a simple means to change the operational range of the device to shorter or longer wavelengths. Fig. 5c shows that diffraction and Au plasmon features shift towards longer wavelengths as the pitch becomes longer, (as would be the case for a dielectric photonic crystal with the same geometry). Figure 5d shows that pyramid size provides control over the intensity and spectral width of the dispersive features. Smaller pyramids result in shallower and sharper peaks. Changing the thickness/refractive index of either the ion gel or spacer layer also induces a shift in the wavelength of the Salisbury screen cavity mode, as well as to phase matching and diffraction modes associated with the pyramid structures.

As shown in Figs 2 and 3 the wavelength of the graphene plasmon resonance is dependent on chemical potential and can be tuned. This in combination with the ability to have complete control over all of the device’s optical properties through optimizing the geometric parameters of the grating structure or the thickness of the spacer or ionic gel layers allows for a flexible range of applications that operate in different spectral ranges.

### Angle of incidence and polarization

So far only the behaviour for waves incident exactly perpendicular to the surface of the device have been examined. The analysis is now extended to look at what happens for any angle of incidence to the surface, again on a step by step basis to aid clarity and show proof of physical processes at play. The angular analysis will focus only on s polarization as its purpose is to further elaborate the physical mechanisms behind the absorption enhancement in graphene. The angular dependence of the absorption spectra of the device for both s and p polarization can be seen in Figure s4 of the supplementary information.

**Figure 6:**
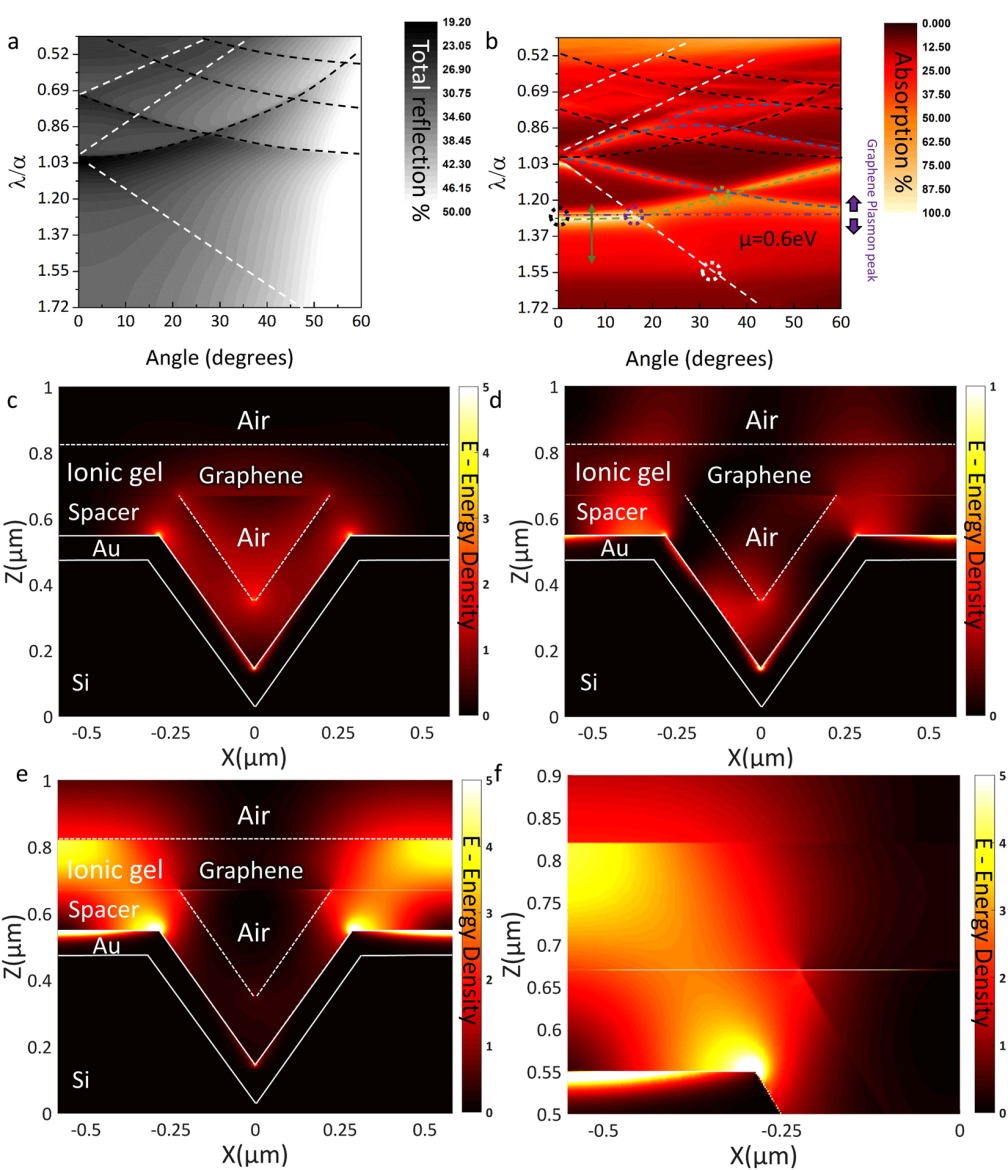
RCWA simulation of the device showing (**a**) total reflection data for a range of photon incident angles plotted versus normalized frequency (wavelength/pitch) when not including the gold back reflector and graphene layer. White dashed lines correspond to 1^st^ order diffractive modes and black dashed lines to dispersive modes associated with the 2-dimensional grating geometry. (**b**) absorption spectra now including the graphene and Au layers. The extra green (fundamental mode) blue and purple lines appearing correspond to Au and Graphene plasmon modes respectively. (**c**–**e**) show FDTD simulation results from the white, green, and purple circle markers in b. respectively. (**f**) Zoomed in version of (**e**) showing that the coupling that occurs between the gold and graphene plasmon modes.

Figure 6a shows a greyscale RCWA simulation mapping spectra for a purely dielectric device without the gold back reflector or graphene layer (*as was the case for* Fig. 5a). X-axis relates to angle of incidence and y-axis scales as λ/α (wavelength/pitch). Putting the analysis in the context of photonic crystals, Fig. 6a effectively plots the ‘photonic band structure’ of the lattice along one symmetry direction (ΓX). In this case the y-axis is proportionate to normalised frequency, and the x-axis relates to k-vector component resolved in the plane of the lattice.

A set of straight and curved diagonal lines with multiple crossing points are observed. Straight lines (*white dashed overlay lines*) correspond to zero and 1^st^ order diffraction^34^, whereas curved lines (*black dashed overlay lines*) correspond to dispersive modes associated with the 2-dimensional grating geometry. Points along these lines correspond to conditions whereby incident light become coupled to the lattice resulting in propagating waves in the lateral direction (in the plane of the lattice). Simple crossing points between bands are observed rather than points of inflection (which would indicate presence of mini stop-bands). This is because the lattice pitch of the underlying structure is relatively large hence photonic band gaps are not observed, just continuous dispersive and diffractive modes. One point to note, no features associated specifically with the Salisbury screen micro-cavity are observed in this situation because without the gold back reflector light mainly passes through the substrate (as shown previously in Fig. 2d), and interaction with the micro-cavity is extremely weak.

Figure 6b shows the equivalent data with the gold reflector and graphene reinstated with the same guide-lines overlaid. As was the case in Fig. 5b a new set of gold plasmon resonances become introduced (green and blue dashed overlay lines), as well as the graphene plasmon band (*purple dash-dot overlay line*) which can be wavelength tuned by adjusting chemical potential. The plasmon bands are much broader than the dielectric dispersion lines (black and white dashed overlay lines). Points of intersection between the graphene and gold plasmon bands correspond to perfect phase matching conditions (*x-axis of this diagram relates to in-plane phase*) allowing efficient transfer of energy between the structures.

Figure 6c–e, show the resulting Field density for different combinations of wavelength and incidence angle. These correspond to the white, green, and purple circle markers on Fig. 6b respectively and are chosen to illustrate conditions for different spectral features of interest and further verify the mechanism behind the increased absorption in the graphene monolayer. (Incidentally field plot Fig. 3b corresponds to the black coloured circle on Fig. 6b).

Figure 6c corresponds to the white circle in Fig. 6b, which lies on a diffractive mode associated with the underlying periodic dielectric structure (diagonal straight white dashed line), and is present irrespective of the gold coating (as it shows up identically on Fig. 6a). Looking at the field distribution in Fig. 6c a strong optical field present in the internal volume of the pit can be seen. No field is present above the top surface between the pits, hence showing that the periodic array of pyramidal pits interacts strongly with incident light via a diffraction process.

Figure 6d corresponds to the green circle in Fig. 6b, which lies on the fundamental gold plasmon band (green dashed line in Fig. 6b), but away from the diffraction band (white dashed diagonal line) and vertical cavity resonance (broad peak marked but the green arrow). The excitation of a surface plasmon located at the top surface of the gold in the spatial regions between the pits can be clearly seen. It is also noted that a significant part of the plasmon field is thrown vertically upwards into the z direction and some of it is located inside the pyramidal pit. The graphene layer is also weakly visible in the field plot showing that a small portion of the field is coupled to the atomic monolayer. It is noted that the graphene layer is not visible in Fig. 6c confirming that a gold surface plasmon mode is required to feed power into the graphene layer.

Inspecting Fig. 6b more closely one can observe that between 0^0^–18^0^ the fundamental (lowest order) plasmon band (dashed green line) does not change wavelength with angle of incidence, has zero gradient, and therefore corresponds to a zero group velocity (stationary) plasmon mode which is localised to the top surface of the gold between the pits. This results in strong single step coupling of incident light over a wide range of surface incidence angles, and allows efficient coupling of light directly to the fundamental plasmon mode from a high NA focussing lens.

By adjusting the chemical potential, the graphene plasmon resonance (purple dash-dot line in Fig. 6b) can be tuned to overlay the stationary plasmon mode (dashed green line in Fig. 6b) over the wide angular range where it is flat, resulting in optimal coupling to the graphene layer itself. It is also noted that the vertical cavity (Salisbury screen) mode overlaps directly and independently provides increased absorption over this region resulting in the wide horizontal white feature of the plot marked by the green arrow.

Figure 6e shows the field distribution corresponding to this point where the diffracted mode (diagonal white line) overlaps with both the graphene plasmon (dash-dot purple line), as well as the fundamental plasmon mode (dashed green line), and the vertical cavity mode (white horizontal feature). Features of the plot are very similar to Fig. 3b (black circle marker in Fig. 6b). Fig. 6f is a zoomed in version of 6.e clearly showing coupling between the gold plasmon mode and the graphene plasmon mode. Overall this energy transfer process between the gold and graphene plasmon mode results in very efficient coupling of incident light to excited plasmons in the atomic monolayer.

### Rapid optical switching

Most significantly, since incident light couples to a doubly-resonant static (non-travelling) wave, it can be predicted that a very small change in chemical potential would rapidly detune the coupling conditions blocking optical energy transfer to the graphene, hence we propose that this design provides basis for fast electro-optical switching which could be exploited in as an optical modulator, or as an optical memory element.

### Tuneable sensors and couplers

Referring back to Fig. 6b, it is noted that all other surface plasmon bands (dashed blue lines in Fig. 6b) are curved. This means that coupling wavelength would change with either applied gate voltage (chemical potential) or angle of incidence, hence under these circumstances plasmon coupling wavelength can be electrically or mechanically tuned. Electrical wavelength tuning of surface plasmons in a practical configuration would be extremely important for chemical sensing methods such as SPR and SERS, and would introduce the possibility of a host of new integrated optical plasmonic devices.

## Conclusion

In conclusion efficient electrically tunable near total optical absorption in a device implementing monolayer graphene is reported. The combination of a two-dimensional diffraction grating with a hybrid gold-insulator-graphene multilayer setup forms a doubly resonant plasmonic structure providing strong absorption enhancement in the graphene film. This enhancement can even provide a 1650% percent of absorption increase in the graphene layer when compared with a device not implementing the proposed hybrid setup. The physical mechanism behind this enhancement is a combination of increased diffraction efficiency for the grating structure, and resonant coupling between plasmons generated in the gold layer to the ones generated in Graphene. The frequency of plasmon excitations in the graphene layer can be controlled electrostatically with the use of an ionic gel layer used in a gate configuration. Tuning is highly efficient allowing for an estimated 1 μm/0.5 V shift of plasmon absorption wavelength and allowing the device to operate at near-infrared frequencies. Furthermore, the absorption peak that is due to graphene plasmons is spectrally narrow and in combination with the efficient electrostatic control can potentially allow for rapid dynamic switching between high and low absorption values. Most importantly, plasmon excitations can be switched off completely by lowering the chemical potential and moving to the inter-band transition region. Finally, a near zero group velocity plasmon mode was found to be excited in the Au layer over a large range of angles thus allowing optimal coupling over a wide range of incidence angles as would for a large NA focusing lens. Crucially, this configuration allows for probing a static non- travelling wave thus potentially providing the basis for fast electro-optical switching to an optical memory element. The device presented in this work has potential in enabling a variety of tuneable nano-photonic devices including sensors, photonic logic gates, optical interconnects, and electro-optical memories.

## Electronic supplementary material

Supplementary information
