# Supplementary material for: Tuneable strong optical absorption in a graphene-insulator-metal hybrid plasmonic device
Source: Sci Rep. 2017 Aug 4;7:7303. doi: 10.1038/s41598-017-07254-0 (PMC5544744; doi:10.1038/s41598-017-07254-0)
Supplement: Supplementary file 1 — Supplementary information [file 41598_2017_7254_MOESM1_ESM.pdf]

Tuneable strong optical absorption in a graphene-insulator-metal hybrid plasmonic device

N. Matthaiakakis[1,2,*], Xingzhao Yan[1], H. Mizuta[1,2] and M.D.B Charlton[1]

[1]*Department of Electronics and Computer Science, University of Southampton, Southampton, SO17 1BJ, United Kingdom*

[2]*School of Materials Science, Japan Advanced Institute of Science and Technology, Ishikawa 923-1292, Japan*

*E-mail: nm3g12@soton.ac.uk

Electrostatically Tuneable permittivity of Graphene.

The effect of electrostatic doping in Graphene can be estimated by using a simple equation that relates gate voltage to carrier concentration in the monolayer: [1,2]

$$n_g = \frac{V_g \varepsilon_0 \varepsilon_r}{e_q d} \quad (1s)$$

Where V_g is the applied voltage, ε_0 and ε_r the permittivity of vacuum and the relative permittivity of the substrate respectively, e_q the electron charge, and d the substrate thickness. The Chemical potential can then be calculated as: [3]

$$\mu = \hbar v_f \sqrt{\pi n_g} \quad (2s)$$

Where ħ is the reduced Planck constant and, v_f Fermi velocity. Taking into account interband and intraband transitions, the complex conductivity of graphene as a function of chemical potential and wavelength can be calculated with the use of Kubo formula:[4]

$$\sigma_{real}(\omega) = \frac{\sigma_0 \mathrm{H}}{2}\left[\tanh\left(\frac{\hbar\omega + 2\mu}{4k_B T}\right) + \tanh\left(\frac{\hbar\omega - 2\mu}{4k_B T}\right)\right] \quad (3as)$$

$$\sigma_{imag}(\omega) = \frac{4\mu\sigma_0}{\hbar\omega\pi}\left(1 - \frac{2\mu^2}{9t^2}\right) - \frac{H\sigma_0}{\pi}\log\frac{|\hbar\omega + 2\mu|}{|\hbar\omega - 2\mu|} \quad (3bs)$$

$$\sigma_{total}(\omega) = \sigma_{real}(\omega) + i\sigma_{imag}(\omega) \quad (3c)$$

Where $\sigma_0 = e_q^{\ 2}/4\hbar$, $H = \left[1 + (\hbar\omega)^2/(36t^2)\right]$, T the temperature, t the hopping parameter (kinetic energy of electrons hopping between atoms), and ω the angular frequency. This formula takes into account the band broadening that occurs in higher energies of the band structure thus extending beyond the Dirac cone approximation. The permittivity of graphene can finally be obtained through the following equation:

$$\varepsilon(\omega) = \varepsilon_\infty + i\frac{\sigma_{total}}{\omega d_g} \quad (4s)$$

where $\varepsilon_\infty = 5.5\varepsilon_0$ is the background permittivity, [5–8] and d_g the thickness of Graphene.

Ionic gel gate

By using equations $n_g = V_g C$ for the carrier density and $\mu = \hbar v_f \sqrt{\pi n_g}$ for the chemical potential (where V_g the gate voltage, C the gate capacitance, and v_f Fermi velocity) the efficiency of the Ionic gel for modulating the Fermi level in Graphene can be estimated and compared to that of conventional dielectrics. Figure s1 compares the modulation efficiency a 20nm thick SiO_2 gate dielectric, a 20nm HfO_2 gate dielectric, and that of an Ionic gel with a capacitance value of 10.7 $\mu F/cm^2$ as obtained from literature [9] (even higher capacitances up to 30 F/cm^2 have been reported on Si wafers with ionic gel gates)[10]. Thin SiO_2 dielectrics suffer from high leakage currents thus high K dielectrics like HfO_2 are typically used for achieving high capacitance and low voltage

operation. The ionic gel is typically functional as a gate capacitance up to 4eV due to the electrochemical window that leads to high leakage currents above a certain threshold. Nevertheless the Ionic gel easily outperforms conventional dielectrics, providing, much higher Fermi level shifts at a very low voltage. This strong modulation efficiency, transparency, and ability to fully modulate isolated or broken Graphene areas over the entire sample area make ionic gel an ideal candidate for a gate material.

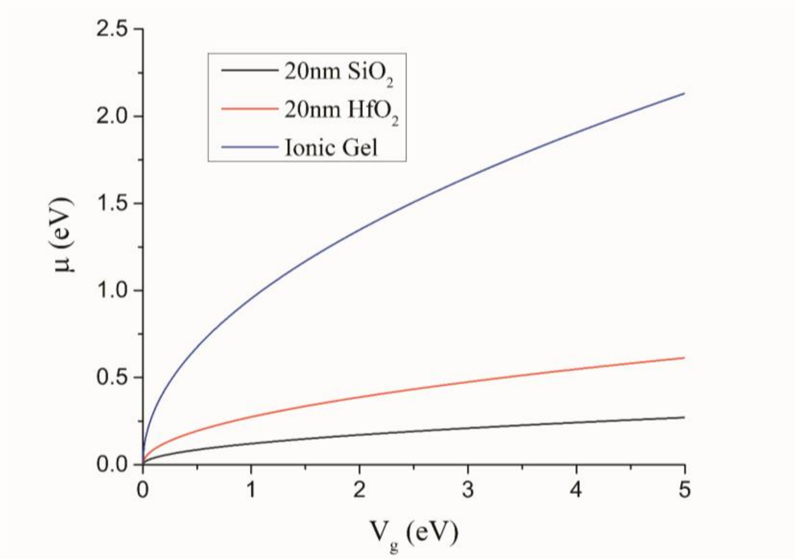

Figure s1. Comparison of chemical potential modulation of Graphene between 20nm thick conventional gate dielectrics (SiO_2 and HfO_2) and an ionic gel with a realistic capacitance value of 10.7μF/cm^2 (from reference [9])

Effect of losses on the graphene layer

Intensity of the plasmon absorption peak strongly depends on optical losses in graphene. These losses are mainly characterized by the imaginary part of permittivity, with higher losses corresponding to broader and lower peaks [11]. Graphene demonstrates significant dissipative losses

in the Terahertz[12] / and infrared optical frequencies |[13] nevertheless recent efforts have demonstrated mobility values in CVD graphene in excess of 350.000 cm^2 V^{-1} s^{-1} at low temperatures and above 50.000 cm^2 V^{-1} s^{-1} at room temperature[14]. Strong modulation over the carrier concentration of graphene while maintaining high mobility rates is still a very strong challenge for the scientific community. Ionic gel gating is the most effective method for achieving high chemical potentials in the graphene layer but can introduce carrier scattering [15–18] . As lower mobility values introduce broader and shallower absorption peaks it is important to investigate at what point the device approaches maximum efficiency. Figure s2 demonstrates the effect of dissipative losses through running RCWA simulations where different mobility values have been used to model the graphene layer. For the ideal case of 30000cm^2 V^{-1} s^{-1} the device operates at a satisfying, over 80%, optical absorption even though the plasmon peak is located dozens of nm away from the central wavelength of enhancement. Down to 10000 cm^2 V^{-1} s^{-1} the device maintains an almost ideal performance with absorption values close to 70%. Nevertheless when the losses in the graphene layer are significant and the mobility becomes lower than 5000 cm^2 V^{-1} s^{-1} the device operation becomes significantly hindered while maintaining a shallow but still notable absorption even at 1500 cm^2 V^{-1} s^{-1}. The design suggested is this study is extremely flexible and gate setups implementing different types of transparent dielectrics can be used for different applications without much change in design given that their refractive index is of a similar value. Applications that require broad band modulation but not extremely high optical absorption can use an ion gel as the gate dielectric while devices that require short range modulation and very strong absorption can implement SiO_2 gates in combination with chemical doping to define a central region for the chemical potential in graphene to electrically tune around. Nevertheless the progress in the field

of graphene is moving at extremely rapid rates and thus higher mobility rates in combination with effective gating methods should be achievable in the future.

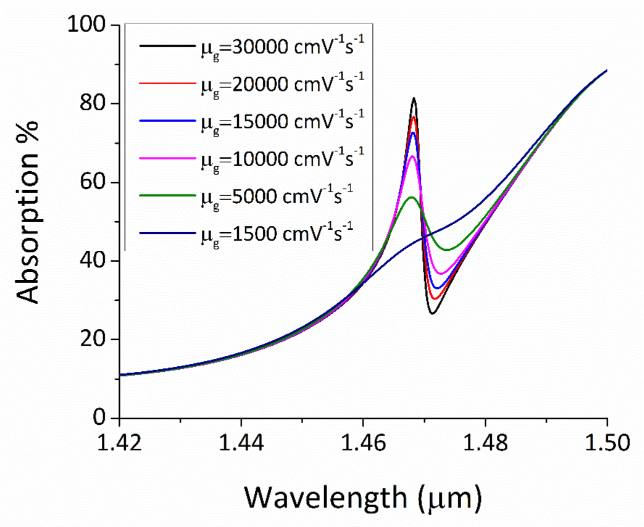

Figure s2. RCWA simulation spectra for different values of mobility for the graphene layer. As the mobility decreases the absorption also becomes much lower and broader.

Salisbury screen

The Salisbury screen[19] was invented in the 1940s as a selective wavelength anti-reflection radar material. Its original implementation consisted of a metal reflector and a graphite absorber layer separated by a transparent dielectric spacer. Similar to a quarter-wave antireflective coating, reflections at the material interfaces destructively interfere to give zero reflection at a specific incident wavelength. Nearly total absorption is achieved when waves reflected from the back reflector and the surface of the absorptive layer have equal amplitude and a phase difference of 180°. In order to achieve strong destructive interference the transparent spacer separating the back reflector from the top absorptive layer must have a thickness:

$$d_s = m\lambda / 4n_s \quad (5s)$$

where d_s the thickness of the spacer and n_s the value of the spacer refractive index, and m is an integer cavity mode number.

Modifying equation 5s to adjust it for a multilayer setup as for the case of our device gives the following conditions for destructive interference in the vertical cavity:

$$\lambda = 4(n_{s1}d_{s1} + n_{s2}d_{s2}) / m \quad (6s)$$

where d_{s1} the thickness of the spacer and n_{s1} the value of the spacer refractive index, d_{s2} the thickness of the ion gel and n_{s2} the refractive index of the ionic gel, and m is an integer cavity mode number.

If the surface layer is not perfectly absorptive, photons get reflected back from the surface and so become recycled until they eventually get absorbed[20].

Contribution of the Salisbury screen to the absorption spectra

It can be seen from figure s3 that the absorption due to the Salisbury screen (peak marked by an S and a white dotted) is spectrally wide and quite low in terms of intensity when compared to the gold and graphene plasmon peaks. The absorption from the Salisbury screen is low because of the existence of the pyramid structures resulting in the majority of the surface not being flat thus providing only a few percent of extra absorption. From the contour profile inset of figure s3 (top) it can be seen that the Salisbury screen does not actually result in increased absorption in the graphene or gold layers, instead it is an isolated effect that increases the absorption additively but independently from the other absorption features that appear in the spectrum. This is easy to see as the absorption is increased for the overall spectral region and the peaks originating from other physical mechanisms are simply shifted upwards but are not increased in intensity. Only when the

graphene plasmon excitation has at least some overlap with the gold plasmon peak, enhanced absorption in the monolayer can be observed.

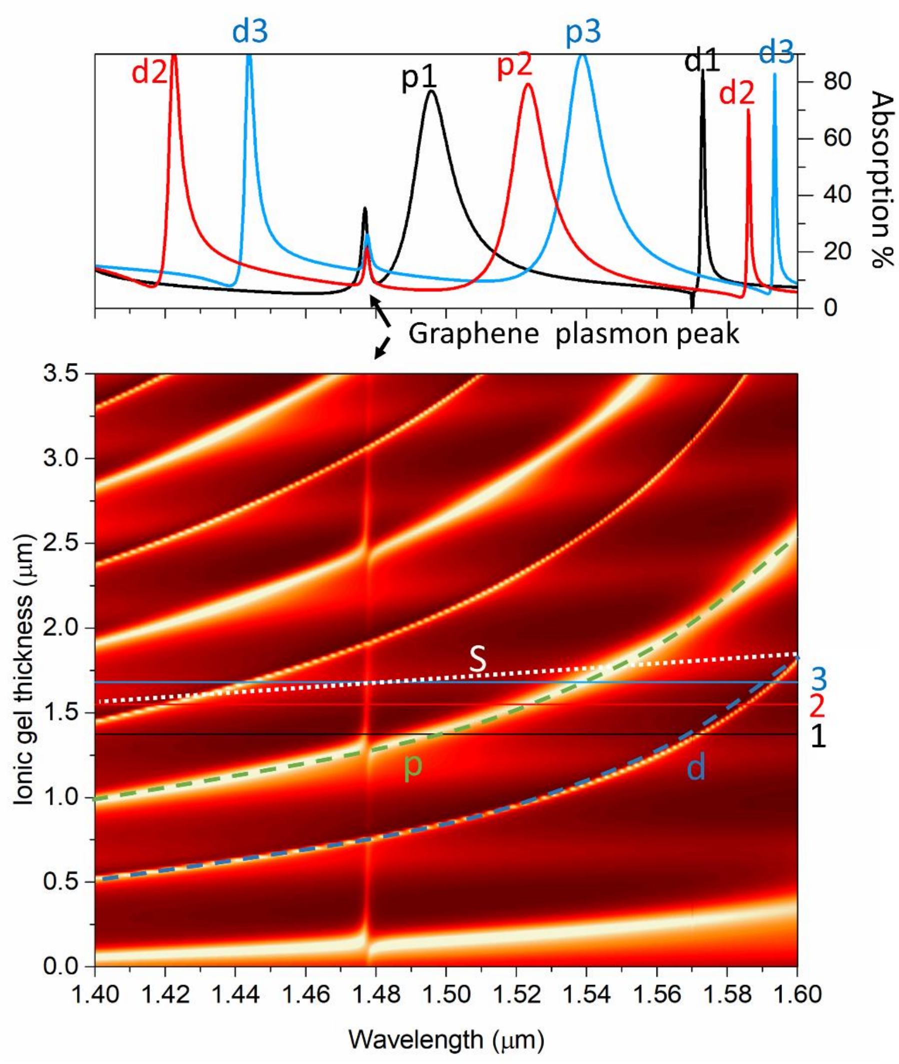

Figure s3. Contour plot and corresponding contour profiles inset (top) of absorption spectra for increasingly thick ionic gel layers. The white dashed line in the contour plot corresponds to the Salisbury screen peak (labelled as S), the green dashed curve line to the gold plasmon peak (labelled as p), and the blue dashed curved line to the diffraction line (labelled as d). The solid black, red, and blue lines labelled as 1,2, and 3 respectively correspond to the contour profiles presented in the inset on top. The graphene peak has been marked with black arrows.

Angle of incidence and polarization

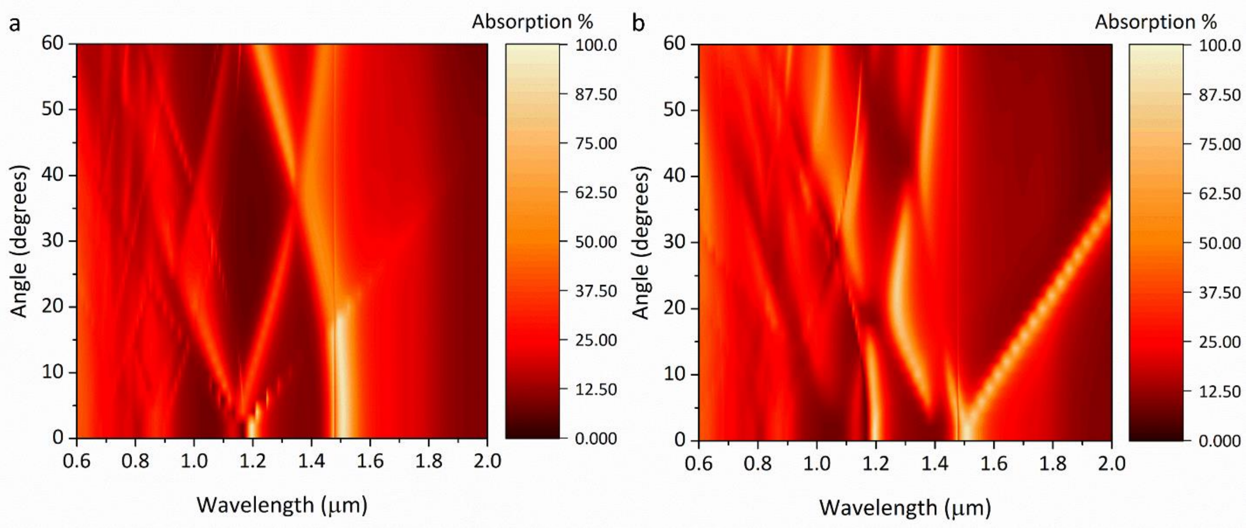

Figure s4. Absorption spectra versus angle of incidence for **a.** s polarization and **b.** p polarization.

References

1. Danaeifar, M., Granpayeh, N., Mohammadi, A. & Setayesh, A. Graphene-based tunable terahertz and infrared band-pass filter. *Appl. Opt.* **52,** E68-72 (2013).
2. Novoselov, K. S. *et al.* Two-dimensional gas of massless Dirac fermions in graphene. *Nature* **438,** 197–200 (2005).
3. Avouris, P. & Freitag, M. Graphene Photonics, Plasmonics, and Optoelectronics. *IEEE J. Sel. Top. Quantum Electron.* **20,** 6000112; DOI:10.1109/JSTQE.2013.2272315 (2014).
4. Stauber, T., Peres, N. M. R. & Geim, a. K. Optical conductivity of graphene in the visible region of the spectrum. *Phys. Rev. B - Condens. Matter Mater. Phys.* **78,** 1–8 (2008).
5. Lin, I. Optical Properties of Graphene from the THz to the Visible Spectral Region. *Thesis* Date of access:12/04/2016 (2012).
6. Klintenberg, M. *et al.* Evolving properties of two-dimensional materials: from graphene to graphite. *J. Phys. Condens. Matter* **21,** 335502; DOI:10.1088/0953-8984/21/33/335502 (2009).
7. Taft, E. & Philipp, H. Optical Properties of Graphite. *Phys. Rev.* **138,** A197–A202 (1965).
8. Johnson, L. G. & Dresselhaus, G. Optical Properties of Graphite. *Phys. Rev. B* **7,** 2275–2285 (1973).
9. Pu, J. *et al.* Highly flexible MoS2 thin-film transistors with ion gel dielectrics. *Nano Lett.* **12,** 4013–7 (2012).
10. Lee, J., Panzer, M. J., He, Y., Lodge, T. P. & Frisbie, C. D. Ion gel gated polymer thin-film transistors. *J. Am. Chem. Soc.* **129,** 4532–4533 (2007).
11. Gao, W., Shu, J., Qiu, C. & Xu, Q. Excitation of plasmonic waves in graphene by guided-mode resonances. *ACS Nano* **6,** 7806–13 (2012).
12. Tassin, P., Koschny, T. & Soukoulis, C. M. Graphene for terahertz applications. *Science (80-.).* **341,** 620–621 (2013).
13. Tassin, P., Koschny, T., Kafesaki, M. & Soukoulis, C. M. A comparison of graphene, superconductors and metals as conductors for metamaterials and plasmonics. **6,** 259–264 (2012).
14. Banszerus, L. *et al.* Ultrahigh-mobility graphene devices from chemical vapor deposition on reusable copper. *Sci. Adv.* **1,** 1–6 (2015).
15. Fang, Z. *et al.* Gated tunability and hybridization of localized plasmons in nanostructured graphene. *ACS Nano* **7,** 2388–2395 (2013).
16. Kim, U. J. *et al.* Modulation of the Dirac Point Voltage of Graphene by Ion-Gel Dielectrics and Its Application to Soft Electronic Devices. *ACS Nano* **9,** 602–611 (2015).
17. Liu, J. *et al.* Enhanced performance of graphene transistor with ion-gel top gate. *Carbon N. Y.* **68,** 480–486 (2014).
18. Lee, S.-K. *et al.* Photo-patternable ion gel-gated graphene transistors and inverters on plastic. *Nanotechnology* **25,** 014002; DOI:10.1088/0957-4484/25/1/014002 (2014).
19. Fante, R. L. & McCormack, M. T. Reflection properties of the Salisbury screen. *IEEE Trans. Antennas Propag.* **36,** 1443–1454 (1988).
20. Thareja, V. *et al.* Electrically Tunable Coherent Optical Absorption in Graphene with Ion Gel. *Nano Lett.* 150211144205005 (2015). doi:10.1021/nl503431d
